# QSAR-guided discovery of novel KRAS inhibitors for lung cancer therapy

**DOI:** 10.3389/fbinf.2025.1663846

**Published:** 2025-11-17

**Authors:** Osasan Stephen Adebayo, George Oche Ambrose, Daramola Olusola, Adefolalu Oluwafemi, Hind A. Alzahrani, Abdulkarim Hasan

**Affiliations:** 1 Department of Laboratory Medicine, Ministry of Health (Prince Mishari Bin Saud Hospital, Baljurashi, Al-Baha), Al-Baha, Saudi Arabia; 2 Department of Pathology, Faculty of Medicine, Al-Azhar University, Cairo, Egypt; 3 University of Ilorin Teaching Hospital, Ilorin, Nigeria; 4 North Devon District Hospital, Barnstaple, United Kingdom; 5 Obafemi Awolowo University Teaching Hospital, Ile-Ife, Osun, Nigeria; 6 Department of Basic Science, College of Applied Medical Sciences, University of Al-Baha, Al-Baha, Saudi Arabia; 7 Department of Medical Laboratory, Al-Baha Health Cluster, Ministry of Health, Al-Baha, Saudi Arabia; 8 Saudi Arabia Board of Preventive Medicine, Al-Baha, Saudi Arabia

**Keywords:** KRAS mutations, QSAR, *de novo* design, GA-MLR model, KRAS inhibitor

## Abstract

**Introduction:**

KRAS mutations are key oncogenic drivers in lung cancer, yet effective pharmacological targeting has remained a major challenge due to the protein's elusive and dynamic binding pockets. Computational modeling offers a promising route to identify novel inhibitors with improved potency and selectivity.

**Methods:**

A quantitative structure–activity relationship (QSAR) modeling approach was developed to predict the inhibitory potency (pIC_50_) of KRAS inhibitors and support *de novo* drug design. Molecular descriptors for 62 inhibitors retrieved from the ChEMBL database (CHEMBL4354832) were computed using Chemopy. Following descriptor normalization and dimensionality reduction, five machine learning algorithm spartial least squares (PLS), random forest (RF), stepwise multiple linear regression (MLR), genetic algorithm optimized MLR (GA-MLR), and XGBoost were applied. Model performance was evaluated using *R* ^2^, RMSE, and MAE, while permutation-based importance and SHAP analyses provided feature interpretability.

**Results:**

Among the models tested, PLS exhibited the best predictive performance (*R* ^2^ = 0.851; RMSE = 0.292), followed by RF (*R* ^2^ = 0.796). The GA-MLR model, based on eight optimized molecular descriptors, achieved good interpretability and robust internal validation (*R* ^2^ = 0.677). Virtual screening of 56 *de novo* designed compounds within the model's applicability domain identified compound C9 with a predicted pIC_50_) of 8.11 as the most promising hit.

**Discussion:**

This integrative QSAR modeling and *de novo* design framework effectively predicted the bioactivity of KRAS inhibitors and facilitated the identification of novel candidate molecules. The findings demonstrate the utility of combining interpretable machine learning models with virtual screening to accelerate the discovery of potent KRAS inhibitors for lung cancer therapy.

## Introduction

Lung cancer is one of the most prevalent malignancies globally and remains the leading cause of cancer-related deaths, responsible for about 1.8 million deaths yearly (Sung et al., 2021). In non-small cell lung cancer (NSCLC), mutations in the Kirsten rat sarcoma viral oncogene homolog (KRAS) gene are among the most frequent genetic alterations, particularly in smokers, and are associated with aggressive tumor phenotypes and resistance to targeted therapies (Pylayeva-Gupta et al., 2011; Westover et al., 2016).

Historically, the development of direct KRAS inhibitors has been challenging due to the protein’s high affinity for GTP/GDP and the absence of easily targetable binding sites, earning it the label of “undruggable” (Cox et al., 2014). However, recent advances in covalent inhibitors, such as those targeting the KRAS G12C mutation (e.g., sotorasib), have demonstrated clinical efficacy and reignited interest in targeting KRAS-driven tumors (Lanman et al., 2019).

Despite this progress, most KRAS mutations beyond G12C remain therapeutically elusive, necessitating the exploration of novel chemical scaffolds and mechanisms of inhibition. Accordingly, a promising avenue for rational drug design is provided by computational methods such as quantitative structure–activity relationship (QSAR) modeling. By using statistical and mathematical techniques to link chemical structure and biological activity, QSAR models make it possible to predict the activity of untested compounds (Tropsha, 2010; Cherkasov et al., 2014). Machine learning and genetic algorithm-enhanced QSAR strategies further enhance model performance by optimizing descriptor selection and minimizing overfitting (Todeschini and Consonni, 2009).

## Materials and methods

### Dataset compilation

A dataset comprising 62 KRAS inhibitors (Supplementary File S1) was retrieved from the ChEMBL database (CHEMBL ID: CHEMBL4354832) (Lanman et al., 2019). The retrieved compounds included their experimentally measured IC50 values (in nM). Each compound was standardized, and duplicates were removed to ensure data consistency. The IC50 values were transformed into pIC50 values using the standard conversion equation:

$$\text{pIC}50=-\log10\left(\text{IC}50\times 10^{-9}\right)$$

This transformation provided a more suitable scale for regression modeling.

### Molecular descriptor calculation

Molecular descriptors were calculated using the ChemoPy package in Python (Cao et al., 2013). Descriptors generated included topological, constitutional, geometrical, and electronic features. The resulting dataset was filtered leaving only numeric descriptors (Supplementary File S2). Columns with missing values and zero variance were excluded. The final descriptor matrix included a diverse set of molecular features relevant to QSAR modeling.

### Preprocessing and feature reduction

Before model training, descriptors were standardized by centering to the mean and scaling to unit variance. Highly correlated descriptors (Pearson’s |r| >0.95) were removed to reduce multicollinearity (Sulaiman et al., 2021). Among the remaining descriptors, the top 50 features with the highest variance were selected for further analysis. The dataset was then split into training (70%) and test (30%) sets using stratified sampling on the pIC50 values.

### Feature selection using genetic algorithm (GA)

A Genetic Algorithm (GA) (Cho et al., 2002) was employed to identify an optimal subset of descriptors that maximized the adjusted R-squared ( $R_{\left\{\text{adj}\right\}}^{2}$ ) while penalizing model complexity. The fitness function used was:

$$\text{Fitness}=R_{\left\{\text{adj}\right\}}^{2}-\frac{k}{n}$$

Where k is the number of selected descriptors and n is the number of training samples. The Genetic Algorithm was run with binary chromosome representation, 50 generations, and 10 consecutive runs without improvement as stopping criteria. The final GA-selected features were used to train a multiple linear regression (MLR) model.

### Model development

To develop robust predictive models for estimating the pIC_50_ values of KRAS inhibitors, multiple machine learning algorithms were employed and benchmarked. The primary model utilized was a Genetic Algorithm-optimized Multiple Linear Regression (GA-MLR) model. In this approach, a binary genetic algorithm was applied to select an optimal subset of molecular descriptors from the training dataset. The fitness function for the GA was designed to maximize the adjusted *R* ^2^ of the resulting linear model while incorporating a penalty term proportional to the number of selected features, thereby discouraging overfitting. The linear model constructed using the selected descriptors followed the general form:

$$y=\beta_{0}+\beta_{1x_{1}}+\beta_{2x_{2}}+\ldots +\beta_{{\text{nx}}_{n}}$$

where y is the predicted pIC_50_, β_0_ is the intercept, β_1_ … βn are the regression coefficients, and x_1_ … x_n_ represent the GA-selected standardized descriptors.

In parallel, a Stepwise Multiple Linear Regression (Stepwise MLR) model was developed using bidirectional stepwise selection based on the Akaike Information Criterion (AIC) (Ghani and Ahmad, 2010). This method involved iterative addition and removal of variables from a null model to a full model, selecting the combination of descriptors that minimized AIC.

For comparative purposes, a Partial Least Squares (PLS) regression model was constructed using the kernel algorithm. The optimal number of latent components was determined through 10-fold cross-validation. Additionally, a Random Forest (RF) model was implemented using 500 trees, leveraging the randomForest package. Feature importance in the RF model was quantified using a permutation-based increase in mean squared error (MSE), enabling interpretation of variable contributions.

Lastly, an Extreme Gradient Boosting (XGBoost) model was trained using the xgboost package with a squared error loss function. The model was tuned with a maximum tree depth of 6, a learning rate (η) of 0.1, and 200 boosting iterations.

Each of these models were trained and validation was achieved using standardized descriptors derived from the 64-compound dataset retrieved from the ChEMBL database (CHEMBL ID: CHEMBL4354832), and their performance metrics were compared using *R* ^2^ and RMSE on both training and test sets.

Model performances were evaluated on the test set using *R* ^2^, RMSE, and MAE.

### SHAP and permutation-based interpretability

In order to interpret feature contributions in the RF model, SHAP values were computed using the iml package in R (Molnar et al., 2018). To determine how each variable affected the predictive power of the model, permutation-based feature importance was also calculated using the same package.

### Domain of applicability (DOA) assessment

Mahalanobis Distance (MD) (Roy et al., 2015) was used to assess whether novel compounds fell within the applicability domain of the training set. The MD was computed as:

$$D2={\left(x-\mu \right)}^{T}\Sigma^{-1}\left(x-\mu \right)$$

Where μ is the mean vector and Σ is the covariance matrix of the normalized training set. A threshold based on the 95th percentile of the χ2 distribution with 8 degrees of freedom was applied. Compounds with MD above this threshold were flagged as outside the DOA.

### Virtual screening and pIC_50_ prediction

To identify novel inhibitors of KRAS, an evolutionary *de novo* design strategy was implemented using the DataWarrior software (López-López et al., 2019). This method seeks to explore vast regions of chemical space by mimicking natural evolution to create new molecules optimized for drug-likeness and target-specific similarity. The process began with a seed molecule (Compound ID: 2363810; Supplementary File S1), chosen for its known KRAS inhibitory profile and desirable drug-like properties. DataWarrior applied random chemical transformations to this initial structure, including atom substitutions, bond rearrangements, and ring modifications, to generate a larger first-generation library of child compounds. These structural mutations were probabilistically biased toward those that improved molecular drug-likeness, as measured by built-in scoring metrics, while chemically implausible modifications were penalized or excluded.

Each resulting compound underwent multi-objective evaluation using fitness criteria such as drug-likeness, pharmacophore alignment, and 3D shape similarity to the reference KRAS inhibitor. Specifically, two similarity scores were used to guide molecular selection: the SkelSpheres similarity score, which captures topological resemblance, and the Flexophore similarity score, which quantifies 3D pharmacophore overlap. Compounds from each generation were ranked based on these criteria, and top performers were retained as parents for the next-generation. Over multiple cycles and generations, the algorithm progressively refined the structures, selecting candidates with fitness scores approaching 1.000 and similarity metrics (both SkelSpheres and Flexophore) often exceeding 0.98.

Ultimately, 56 molecules were selected across seven generations (Supplementary File S3), encompassing diverse yet drug-like scaffolds. Each compound’s SMILES structure was exported, and the same eight GA-selected descriptors—TASA, RDFE14, grav, RDFE19, PNSA3, RDFM11, RDFP9, and RDFV14—were computed using the Chemopy Python library. These descriptors were normalized using the mean and standard deviation values derived from the training set, ensuring compatibility with the original model space.

The normalized descriptors were then passed into the GA-optimized multiple linear regression (GA-MLR) model, which had been trained on 62 experimental KRAS inhibitors (CHEMBL4354832 dataset). Predicted pIC_50_ values were calculated for all 56 *de novo* compounds. To ensure model applicability, Mahalanobis distance was computed for each prediction, and compounds falling outside the 95% confidence ellipsoid of the descriptor space were flagged as “Outside DOA” (domain of applicability).

This virtual screening workflow successfully yielded novel candidate molecules with promising inhibitory activity. Several of these compounds exceeded a pIC_50_ threshold of 8.0—comparable or superior to reference KRAS inhibitors—and were designated as hits for further consideration. These candidates will be structurally highlighted in the manuscript and considered for *in silico* ADMET profiling and docking-based validation.

### Molecular docking

For molecular docking, we used the KRAS G12 crystal structure (PDB ID: 6CU6 as the receptor. The protein was prepared in BIOVIA Discovery Studio Visualizer and PyMOL by removing crystallographic waters/ions (except those required for structural integrity), retaining the co-crystallized ligand for redocking qualification, adding polar hydrogens, and assigning atom types; Cys12 was kept in its standard form because we modeled noncovalent pre-reaction poses (no covalent bond formation). Ligands (*de novo* designs and C9) were generated in DataWarrior, sanitized, assigned protonation states appropriate for ∼ physiological pH, and energy-minimized; final docking inputs were prepared via PyRx (Open Babel) with Gasteiger charges and PDBQT conversion. Docking was carried out in PyRx/AutoDock Vina, using a search box centered on the Switch-II pocket around the co-crystal pose to fully encompass the H95–Y96–Q99 cryptic region and the Cys12 vicinity; default Vina parameters were used unless otherwise stated. The protocol was qualified by redocking the co-crystallized ligand and visually assessing recovery of the pose and key contacts. For all compounds, multiple poses were generated and ranked by Vina score (kcal·mol^−1^); the top-scoring, clash-free pose consistent with known SII-P pharmacology was retained for analysis. Interaction fingerprints (H-bonding, electrostatics, hydrophobics/π) were inspected and illustrated in Discovery Studio and PyMOL, and binding energies reported from Vina are presented as negative values (more negative = more favorable).

### Software environment

All data preprocessing, modeling, and visualization were conducted in R version 4.3.2. Libraries used include: caret, randomForest, xgboost, iml, GA, pls, Metrics, and ggplot2. The R script used for the analysis is provided as an additional file for reference (Additional file).

## Results and discussion

### Overview of model performance

Five machine learning and statistical regression models were developed to predict the pIC_50_ values of compounds using molecular descriptors generated via Chemopy. These models included: (1) Genetic Algorithm-optimized Multiple Linear Regression (GA-MLR), (2) Stepwise MLR, (3) Partial Least Squares (PLS) Regression, (4) Random Forest (RF), and (5) XGBoost regression. Each model was trained on a dataset consisting of 70% of the observations and validated using the remaining 30%. The evaluation metrics used were the coefficient of determination (*R* ^2^), root mean square error (RMSE), and mean absolute error (MAE).

Table 1 presents the validation performance of each model. The PLS model achieved the highest predictive accuracy with an *R* ^2^ of 0.851 and the lowest RMSE (0.292). The Random Forest model yielded an *R* ^2^ of 0.796 and an RMSE of 0.343. XGBoost achieved *R* ^2^ = 0.688 and RMSE = 0.478. The GA-MLR and Stepwise MLR models yielded comparable *R* ^2^ values of 0.677 and 0.685, respectively, though the Stepwise model demonstrated a slightly lower RMSE (0.590 vs. 0.663).

**TABLE 1:** Summary of validation metrics for each predictive model.

Model	*R* ^2^	RMSE	MAE
GA-MLR	0.677	0.663	0.509
Stepwise MLR	0.685	0.590	0.466
PLS	0.851	0.292	0.218
Random Forest	0.796	0.343	0.255
XGBoost	0.688	0.478	0.379

External-test benchmarking on the standardized pipeline confirms this ranking: PLS (*R* ^2^ = 0.893, RMSE = 0.309), RF (*R* ^2^ = 0.838, RMSE = 0.330), GA-MLR (*R* ^2^ = 0.599, RMSE = 0.518), and XGBoost (*R* ^2^ = 0.439, RMSE = 0.524). We therefore retain GA-MLR as the primary, interpretable model for SAR extraction, while reporting PLS/RF as higher-capacity baselines for context (Table 2).

**TABLE 2:** Summary of validation metrics for each predictive model (external set).

Model	R_squared	RMSE
GA-MLR (final)	0.599	0.518
PLS	0.893	0.309
Random Forest	0.838	0.330
XGBoost	0.439	0.524

To quantify statistical robustness, repeated 10 × 5-fold cross-validation on the training set produced mean *R* ^2^ = 0.370 with 95% quantiles 0.014–0.905, and mean RMSE = 1.02 with 95% quantiles 0.365–1.80. The observed spread is expected for a small, congeneric series and is reflected in the external performance variance.

### Predictive equation from GA-optimized MLR

The GA-MLR model selected eight features using a genetic algorithm that maximized the adjusted *R* ^2^ while penalizing model complexity. The regression equation derived is as follows:

y=6.6791−0.5139·TASA+0.8145·RDFE14+0.6494·grav+0.00898·RDFE19+0.3118·PNSA3−0.3224·RDFM11+0.4943·RDFP9−0.4546·RDFV14

This model yielded an *R* ^2^ of 0.677 and RMSE of 0.663 on the test set, with predicted pIC_50_ values ranging from 5.23 to 9.26.

Using the final GA-MLR (GA + VIF) implementation, N_train = 46, N_test = 16, p = 15 descriptors, with R^2^_train = 0.721, RMSE_train = 0.476, and R^2^_pred = 0.599, RMSE_test = 0.518. We additionally report 95% coefficient confidence intervals for transparency; four coefficients (two positive, two negative) do not cross zero, supporting mechanistic interpretability (Table 3).

**TABLE 3:** Summary of training/test sample sizes (N_train, N_test), descriptor count (p), in-sample fit (*R* ^2^_train, RMSE_train), external test performance (*R* ^2^_pred, RMSE_test), repeated 10 × 5-fold CV statistics (mean *R* ^2^ with 2.5%–97.5% quantiles), and Williams applicability-domain (AD) parameters (train/test coverage; threshold h\*h^\*).

Metric	Value
N_train	46
N_test	16
p	15
R2_train	0.721
RMSE_train	0.476
R2_pred (external)	0.599
RMSE_test	0.518
R2_cv_mean	0.370
R2_cv_2.5%	0.0136
R2_cv_97.5%	0.905
AD_coverage_train	0.742
AD_coverage_test	0.242
h_star	1.043

All metrics are reported in pIC_50_ units where applicable.

### Predictors selected by stepwise MLR

The stepwise MLR model selected 14 descriptors through a bidirectional selection process. Descriptors like RDFE10, RDFM9, WNSA1, RDFE21, and MoRSEE1 were included in the final model equation. On the validation set, the final formula produced an *R* ^2^ of 0.685 and an RMSE of 0.590.

For parsimony, we also derived a BIC-penalized refit from the GA-VIF pool (p = 6), yielding R^2^_pred = 0.429, RMSE_test = 0.632. While less accurate, the BIC model offers a compact explanatory scaffold and can expand the reliable applicability domain in prospective use (Table 4).

**TABLE 4:** Test-set performance and AD coverage for the BIC-selected linear model with fewer descriptors (p).

Model	p	R2_pred	RMSE_test	AD_train_cov	AD_test_cov	N_insideAD
BIC-MLR	6	0.429	0.632	0.726	0.242	15

Where available, inside-AD, test metrics are provided to indicate reliability within the modeled subspace.

### PLS and ensemble models

With two latent components, the PLS regression model produced better predictive results (*R* ^2^ = 0.851; RMSE = 0.292). The Random Forest model, constructed with 500 trees and 16 variables tried at each split, achieved an *R* ^2^ of 0.796 and an RMSE of 0.343. XGBoost, trained using the GA-selected features and tuned with 200 boosting rounds (max depth = 6, eta = 0.1), achieved *R* ^2^ = 0.688 and RMSE = 0.478.

**FIGURE 1:**
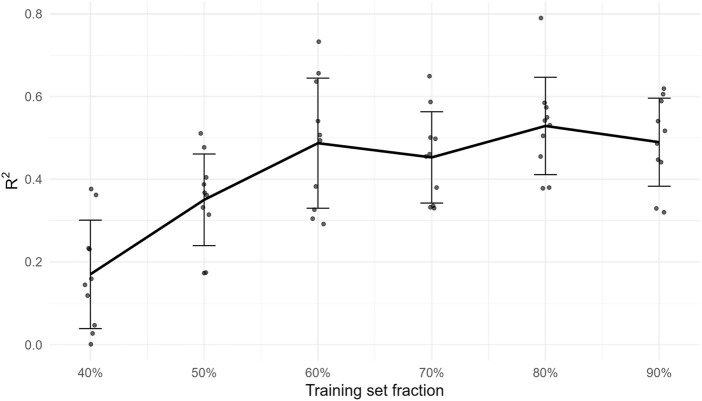
External *R* ^2^ as a function of training-set fraction (20%–90%). Points are replicate subsamples; the solid line is the mean and error bars denote ±1 SD. The plateau indicates diminishing returns at the current data size for this congeneric series.

Under the standardized feature/normalization protocol, PLS improved to *R* ^2^ = 0.893, RMSE = 0.309; RF to *R* ^2^ = 0.838, RMSE = 0.330; XGBoost performed at *R* ^2^ = 0.439, RMSE = 0.524. These baselines bound the achievable accuracy and corroborate that the activity signal is learnable while GA-MLR remains the SAR workhorse (Figure 1).

### Feature importance and model interpretability

**FIGURE 2:**
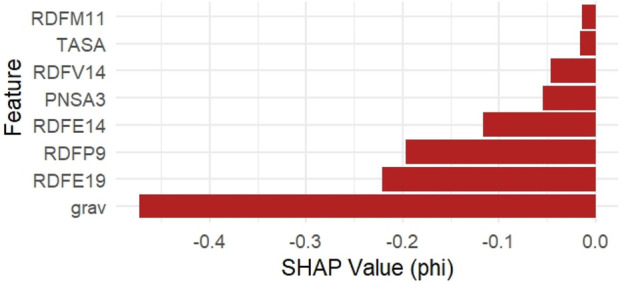
Top 8 permutation-based feature importances from the Random Forest model.

Grav, RDFE19, RDFP9, and RDFE14 were among the most significant descriptors, according to a permutation-based importance analysis employing the Random Forest model (Figure 2). When permuted, these features displayed the largest increase in mean squared error (MSE), suggesting their significance for the predictive performance of the model.

**FIGURE 3:**
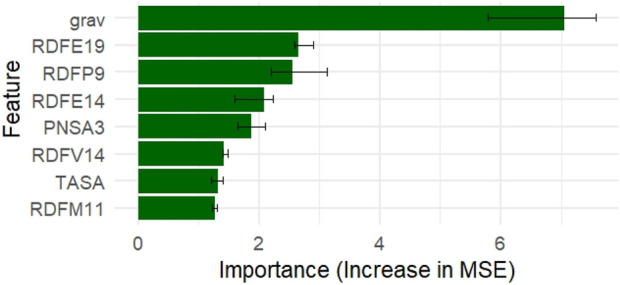
Top 8 SHAP contributions computed using the Random Forest model.

Additionally, SHAP (Shapley Additive Explanations) values were computed using the iml package to provide a local explanation for model predictions. Figure 3 shows the top 8 features ranked by average absolute SHAP values. The grav descriptor exhibited the highest negative SHAP impact on pIC_50_ predictions, followed by RDFE19 and RDFP9.

Descriptor directions inferred from GA-MLR (e.g., positive contributions for vdW/shape-weighted RDF/MoRSE families and balanced positive polar surface area) align with the amphiphilic character of the Switch-II pocket and informed substituent vectors subsequently tested by docking.

### Model visualization and residual analysis

**FIGURE 4:**
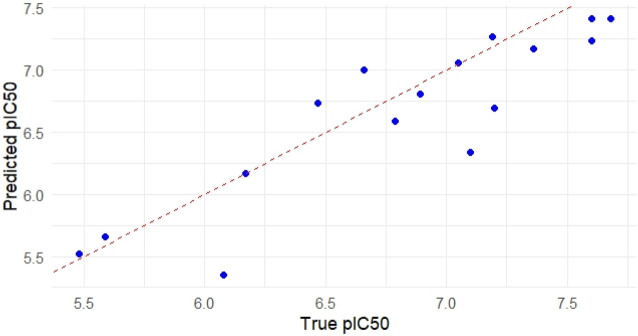
Performance and interpretability of the Random Forest (RF) model.

Visualization techniques were used to further assess the Random Forest (RF) model’s performance and interpretability. The RF model’s predicted and observed pIC_50_ values are shown in Figure 4. Good predictive agreement is suggested by the scatter plot’s strong alignment along the diagonal reference line (slope = 1). The model’s resilience on the test set is further supported by the fact that the majority of the points cluster around the diagonal with little deviation. This visual coherence supports the previously reported *R* ^2^ of 0.796 and RMSE of 0.343, demonstrating the RF model’s capability to capture variance in the bioactivity dataset.

In addition, feature interpretability was examined using the mean decrease in accuracy metric, as shown in Supplementary Figure S1 (Supplementary File S4). The bar chart ranks the molecular descriptors based on their contribution to model performance. Among the top-ranking descriptors, grav (gravitational index), RDFM4, RDFE5, and RDFU4 demonstrated the highest importance in explaining model variance. These features reflect various physicochemical and 3D spatial properties essential in determining compound-target interaction. Interestingly, features such as RDFM11, RDFV16, and TASA, although initially selected in other models, were assigned relatively low importance in the RF model, which may suggest non-linearity or redundancy under ensemble learning paradigms.

We further quantified uncertainty with split conformal prediction (80/20 proper-train/calibration), achieving 93.8% empirical coverage at a 90% nominal target and a mean prediction-interval width of 5.55 pIC_50_ units, providing calibrated error bars for decision-making.

### Comparative analysis of GA-MLR and stepwise MLR coefficients

To assess the alignment and divergence in feature selection strategies, the multiple linear regression (MLR) models derived via Genetic Algorithm (GA) and Stepwise regression were systematically compared. Table 5 presents the coefficient estimates and associated p-values for predictors retained in both models.

**TABLE 5:** Comparison of GA-MLR vs. Stepwise MLR coefficients.

Variable	Estimate_GA	p_GA	Estimate_stepwise	p_stepwise
(Intercept)	6.6791	1.75E-49	6.6791	6.46E-46
TASA	−0.5139	1.52E-05	−0.3092	0.00028
RDFE14	0.8145	0.00016	0.2550	0.01463
grav	0.6494	0.00033	-	-
RDFE19	0.0090	0.92763	-	-
PNSA3	0.3118	0.01231	-	-
RDFM11	−0.3224	0.00040	−0.3894	0.00316
RDFP9	0.4943	6.81E-05	-	-
RDFV14	−0.4546	0.01444	-	-
RDFE10	-	-	0.3250	0.00213
RDFM9	-	-	0.3570	0.00039
WNSA1	-	-	−0.2156	0.00062
RDFU9	-	-	0.5351	0.00040
RDFM6	-	-	0.1926	0.05153
RDFU4	-	-	−0.3821	0.00331
RDFE21	-	-	−0.1618	0.02145
MoRSEE1	-	-	−0.1429	0.05268
RDFE20	-	-	0.1307	0.10460
RDFV11	-	-	0.1986	0.16125
RDFV16	-	-	0.0961	0.23464

The GA-MLR model selected nine descriptors, including TASA, RDFE14, grav, RDFE19, PNSA3, RDFM11, RDFP9, and RDFV14. Notably, grav, RDFP9, and RDFV14 were exclusively retained by GA, suggesting that these features may offer predictive value in non-sequential optimization processes but were excluded during stepwise elimination due to collinearity or marginal gain in explanatory power.

Conversely, the Stepwise MLR model retained 14 descriptors, including RDFE10, RDFM9, WNSA1, RDFU9, and others not selected by the GA-based approach. Despite some overlap, such as the inclusion of TASA, RDFE14, and RDFM11 in both models, their estimated coefficients and significance levels varied. For instance, TASA was more negatively weighted in the GA model (β = −0.514, p < 0.0001) than in the stepwise model (β = −0.309, p = 0.0003), highlighting differences in the model optimization trajectory.

Overall, the comparative findings highlight the fact that although both feature selection methods find core descriptors that significantly affect pIC_50_, GA might give preference to a smaller group of important variables. Stepwise regression, on the other hand, encourages a more complex model that is guided by small gains in model fit.

### GA-MLR prediction accuracy and coefficient interpretation

**FIGURE 5:**
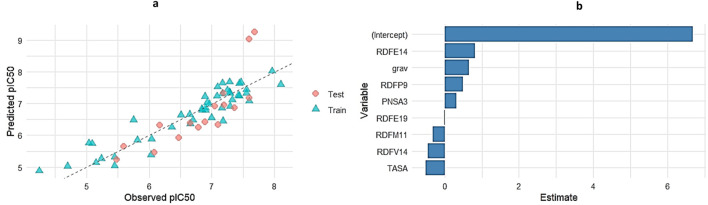
**(a)** Observed vs. Predicted pIC50 values for GA-MLR across training and test sets. **(b)** Regression coefficients for the GA-selected molecular descriptors in the MLR model.

To evaluate the predictive performance of the Genetic Algorithm–based Multiple Linear Regression (GA-MLR) model, a combined visualization of the observed versus predicted pIC50 values for both training and test datasets was generated (Figure 5a). While the predictions for the test set were somewhat more scattered, they generally matched the observed values, while the training set’s points clustered closely along the identity line, suggesting a strong model fit. This indicates that the GA-MLR model was able to strike a fair balance between generalization and training fit.

In addition to visual assessment, coefficient estimates for the selected descriptors were extracted and plotted (Figure 5b). Here, positive coefficients (e.g., RDFE14, grav, PNSA3, RDFP9) indicate descriptors that positively influence pIC50, whereas negative coefficients (e.g., TASA, RDFM11, RDFV14) suggest inhibitory contributions. Notably, RDFE14 had the highest positive impact on pIC50 prediction among the molecular descriptors, while TASA showed the most pronounced negative contribution. The regression model captures the multifaceted nature of the molecular features that drive bioactivity, as demonstrated by this balance of influences.

To guard against chance correlation, Y-randomization with B = 50 label shuffles yielded an empirical p-value ≈0.000, indicating the model captures non-random SAR signal.

### Virtual screening of *de novo* KRAS-designed compounds

A virtual screening of 58 novel compounds created from scratch using DataWarrior was done to investigate the predictive potential of the GA-optimized MLR model. These compounds were structurally inspired by known KRAS inhibitors used in the development of the model. Molecular descriptors were calculated for each compound, and normalization was applied using the training set mean and standard deviation for each of the eight GA-selected features (TASA, RDFE14, grav, RDFE19, PNSA3, RDFM11, RDFP9, RDFV14).

The trained GA-MLR model was then used to compute predicted pIC50 values for the new compounds. The predicted activities ranged from 6.42 to 9.05, suggesting a moderate to high potential for KRAS inhibition. To ensure model reliability, the Mahalanobis distance was computed for each compound, assessing its proximity to the descriptor space defined by the training set.

**FIGURE 6:**
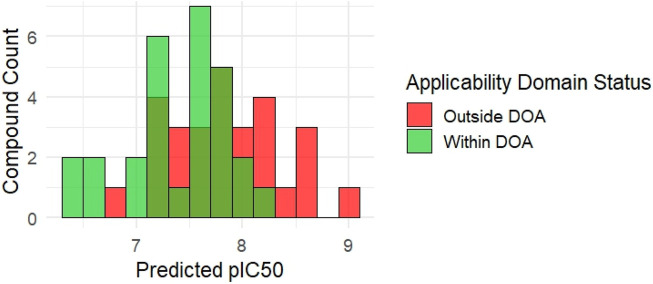
Distribution of predicted pIC50 for 58 *de novo* synthesized KRAS-like compounds. Bars are color-coded to indicate domain of applicability status based on Mahalanobis distance (cutoff = 15).

Figure 6 illustrates the distribution of predicted pIC50 values and highlights compounds flagged as outside the model’s applicability domain (DOA >15.0). Of the 58 molecules, 36 compounds (62%) were classified as within the model’s applicability domain, while 22 compounds (38%) were flagged as extrapolations (outside DOA) (Supplementary File S5). These excluded molecules, despite having favorable predicted pIC50, may require further experimental validation due to structural dissimilarity or feature deviations.

**FIGURE 7:**
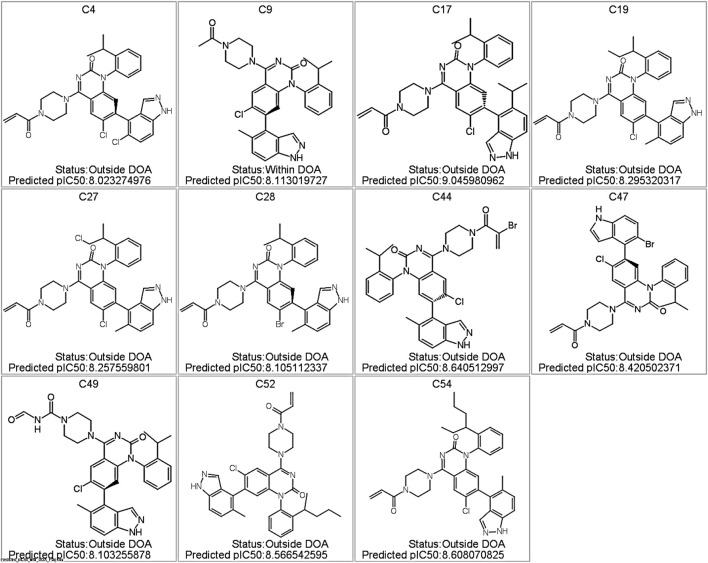
Predicted KRAS Inhibitor Hits with pIC_50_ >8.0 from Virtual Screening of *De Novo* Compounds. Structures of the top-scoring compounds generated via *de novo* design and screened using the GA-optimized MLR model. Only compound C9 was predicted to fall within the applicability domain (DOA); all others are flagged as outside the DOA. Predicted pIC_50_ values are indicated for each compound.

Among the 58 *de novo* synthesized compounds evaluated through virtual screening, several exhibited predicted pIC_50_ values exceeding the benchmark of 8.0, which was used as a reference based on the potency of known experimental KRAS inhibitors. Specifically, compounds C4, C9, C19, C27, C28, C40, C44, C47, C49, C52, and C54 demonstrated predicted pIC_50_ values ranging from 8.02 to 8.64 (Figure 7), marking them as potential virtual hits for KRAS inhibition. Notably, of these, only compound C9 (pIC_50_ = 8.11) was classified as within the model’s applicability domain (DOA), reinforcing its reliability for further consideration. The remaining high-activity compounds, although flagged as being outside the applicability domain, may represent structurally novel scaffolds that warrant experimental validation.

To support generalization claims when chronology is unavailable, we performed leave-cluster-out validation, holding out whole descriptor clusters. Across folds, the mean R^2^ ≈ 0.363 and mean RMSE ≈1.48 (k = 4), penalizing neighborhood overfitting and approximating “novel-analog” performance (Table 6).

**TABLE 6:** For each k-means cluster held out in turn, the table lists training/test sizes and external performance (*R* ^2^, RMSE).

Cluster	N_train	N_test	R_squared	RMSE
1	37	25	0.1005769	0.9484648
2	60	2	1.0000000	2.7320567
3	42	20	0.0985520	0.9912530
4	47	15	0.2545580	1.2480888

LCO, penalizes neighborhood overfitting and approximates generalization to “novel” analog clusters.

### Drug-likeness and toxicity profile

**FIGURE 8:**
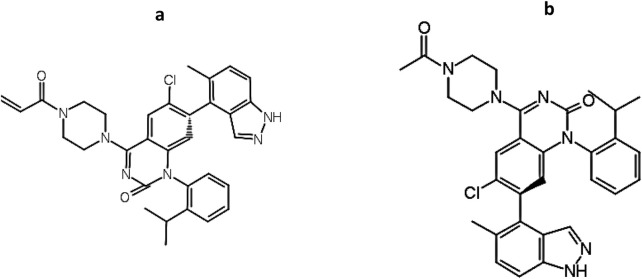
Structural resemblance between **(a)** KRAS inhibitor (Compound ID: 2363810; Supplementary File S1; pIC_50_ = 8.10) and **(b)** C9 (pIC_50_ = 8.11).

To evaluate the drug-likeness of the identified hit compound, ADMET-related physicochemical and toxicity parameters were assessed for compound C9 using DataWarrior software, which is the only *de novo* molecule that both exceeded the benchmark pIC_50_ value of 8.0 and fell within the model’s applicability domain (Figure 8). As shown in Table 7, compound C9 exhibited a predicted pIC_50_ of 8.11, which is slightly higher than that of the known KRAS inhibitor (pIC_50_ = 8.10). Its molecular weight (555.08 Da), cLogP (5.96), number of hydrogen bond acceptors (8), and number of hydrogen bond donors (1) were all comparable to those of the reference compound. The total surface area (410.08 Å^2^) and polar surface area (84.9 Å^2^) further supported its bioavailability profile.

**TABLE 7:** Comparison of physicochemical and toxicity properties between C9 and reference KRAS inhibitor.

Compound ID	pIC_50_	Mol. weight (Da)	cLogP	H-acceptors	H-donors	Total surface area (Å^2^)	Polar surface area (Å^2^)	Mutagenic	Tumorigenic	Reproductive effective
KRAS inhibitor	8.10	567.09	6.22	8	1	422.36	84.9	High	Low	Low
C9 (Hit compound)	8.11	555.08	5.96	8	1	410.08	84.9	None	None	None

Importantly, In-silico toxicity predictions revealed that compound C9 was classified as non-mutagenic, non-tumorigenic, and lacking reproductive toxicity, unlike the reference KRAS inhibitor, which was flagged for high mutagenic potential. This suggests that C9 may represent a safer lead candidate for further preclinical investigation.

**FIGURE 9:**
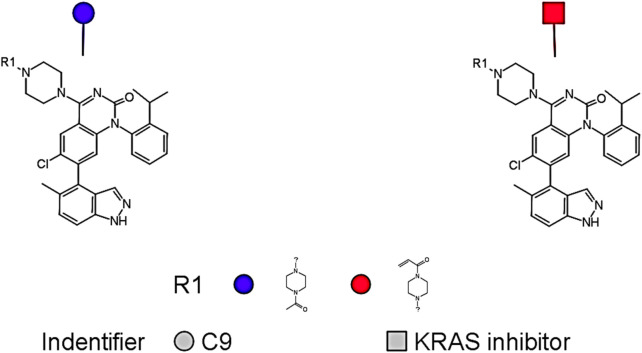
A conserved quinazoline-based scaffold seen in both C9 and Compound ID: 2363810; (Supplementary File S1).

In addition to its favorable ADMET profile, compound C9 exhibited a close structural resemblance to the reference KRAS inhibitor, with subtle modifications at the R1 position. As depicted in Figure 9, both molecules share a conserved quinazoline-based scaffold, which is crucial for KRAS inhibitory activity. However, C9 features a 4-methylpiperidin-4-ylacetamide moiety at the R1 position, which distinguishes it from the acrylamide-bearing R1 group in the reference inhibitor. This modification may contribute to C9’s improved predicted potency (pIC_50_ = 8.11) and non-toxic profile. The simplified side chain in C9 potentially reduces electrophilic reactivity, contributing to its favorable mutagenicity and tumorigenicity scores. These findings suggest that C9 retains the pharmacophoric integrity of the KRAS inhibitor while presenting a safer and equally potent alternative for further lead optimization.

**FIGURE 10:**
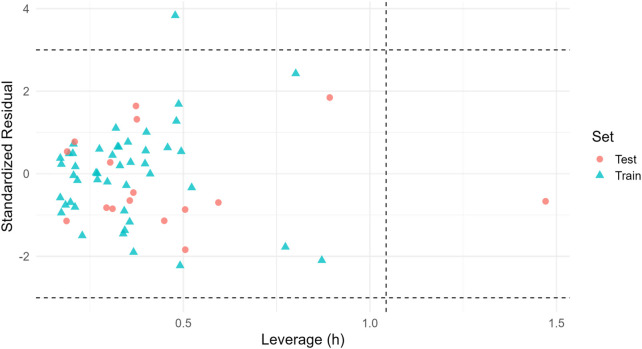
Williams plot (applicability domain). Standardized residuals vs. leverage for train/test (final GA-MLR). Dashed lines: ±3 residuals (horizontal) and h* (vertical). Inside-AD points indicate the most reliable predictions; coverage in Table 8.

Applicability-domain stratification based on Williams leverage (threshold h* = 1.04) showed 74.2% coverage for training and 24.2% for test; we therefore prioritize inside-AD candidates (e.g., C9) for experimental follow-up (Figure 10; Table 8).

**TABLE 8:** External test *R* ^2^ and RMSE reported for the full test set (“All_Test”) and, where available, for inside-AD and outside-AD strata.

Stratum	*R* ^2^	RMSE	N
All_test	0.5988135	0.518295	16
Inside_AD			15
Outside_AD			1

N indicates the number of compounds per stratum. The Williams threshold is computed from the training design matrix.

### Molecular docking of C9 in the KRAS G12 binding pocket

**FIGURE 11:**
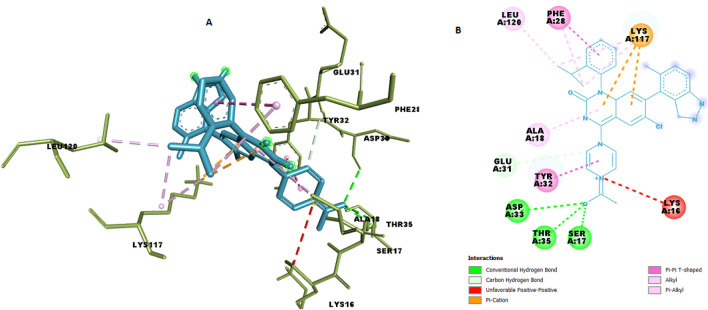
Chemical interaction of compound C9 within the KRAS G12 binding pocket **(a)** 3D interactions and **(b)** 2D interaction.

We docked the top-ranked *de novo* hit C9 into the KRAS G12 binding pocket and benchmarked the protocol against the co-crystallized reference ligand. C9 achieved a docking score of −9.6 kcal mol^−1^, indicative of strong non-covalent complementarity within the pocket. The predicted pose forms a small hydrogen-bond network (ca. 2.26–2.93 Å) with SER17 (HG→O), THR35 (HN→O; OG1···O) and ASP33 (O···O), supported by electrostatic contacts to LYS117 (NZ) and a hydrophobic shell involving PHE28, TYR32, LYS117, LEU120, and ALA18 (π–π and alkyl contacts, ∼3.5–5.1 Å). Representative 3D/2D interaction depictions and per-contact distances are shown in Figure 11 and Table 9.

**TABLE 9:** Details of chemical interactions of C9 with KRAS G12 binding pocket.

Name	XYZ:X	XYZ:Y	XYZ:Z	Distance	Category	From	From chemistry	To	To chemistry
A:SER17:HG - N:UNK1:O	6.0305	27.823	12.966	2.26169	Hydrogen bond	A:SER17:HG	H-donor	N:UNK1:O	H-acceptor
A:THR35:HN - N:UNK1:O	7.233	26.2585	12.0425	2.79979	Hydrogen bond	A:THR35:HN	H-donor	N:UNK1:O	H-acceptor
N:UNK1:O - A:ASP33:O	6.093	27.275	11.0135	2.92842	Hydrogen bond	N:UNK1:O	H-donor	A:ASP33:O	H-acceptor
N:UNK1:O - A:THR35:OG1	7.0485	27.4675	12.7705	2.5282	Hydrogen bond	N:UNK1:O	H-donor	A:THR35:OG1	H-acceptor
N:UNK1:C - A:GLU31:O	0.997	27.2265	10.335	3.73123	Hydrogen bond	N:UNK1:C	H-donor	A:GLU31:O	H-acceptor
A:LYS117:NZ - N:UNK1	−1.92625	23.2282	13.2429	4.35725	Electrostatic	A:LYS117:NZ	Positive	N:UNK1	Pi-orbitals
A:LYS117:NZ - N:UNK1	−1.77083	22.1195	12.7876	3.51304	Electrostatic	A:LYS117:NZ	Positive	N:UNK1	Pi-orbitals
A:PHE28 - N:UNK1	−5.62917	27.4446	12.3228	4.95864	Hydrophobic	A:PHE28	Pi-orbitals	N:UNK1	Pi-orbitals
A:TYR32 - N:UNK1	3.148	24.2891	11.0662	4.36469	Hydrophobic	A:TYR32	Pi-orbitals	N:UNK1	Pi-orbitals
N:UNK1:C - A:LYS117	−4.74833	23.8055	16.4143	3.79833	Hydrophobic	N:UNK1:C	Alkyl	A:LYS117	Alkyl
N:UNK1:C - A:LYS117	−5.66283	23.0475	15.9958	3.63672	Hydrophobic	N:UNK1:C	Alkyl	A:LYS117	Alkyl
N:UNK1:C - A:LEU120	−7.78325	22.4312	15.0076	3.93632	Hydrophobic	N:UNK1:C	Alkyl	A:LEU120	Alkyl
A:PHE28 - N:UNK1:C	−5.26192	27.6692	14.4117	5.00562	Hydrophobic	A:PHE28	Pi-orbitals	N:UNK1:C	Alkyl
N:UNK1 - A:ALA18	−1.22125	27.1132	13.7384	5.10359	Hydrophobic	N:UNK1	Pi-orbitals	A:ALA18	Alkyl

**FIGURE 12:**
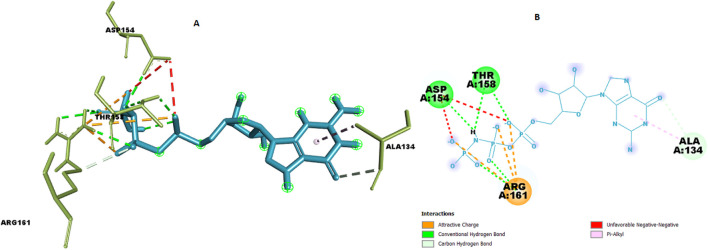
Chemical interaction of co-crystalized compound within the KRAS G12 binding pocket **(a)** 3D interactions and **(b)** 2D interaction.

For the co-crystallized ligand, the docking reproduced a dense polar network dominated by ARG161 (NH1/HH···O)* and THR158/ASP154 hydrogen bonds (≈2.29–3.30 Å), with hydrophobic stabilization at ALA134 (Figure 12; Table 10). This provides an internal control for the docking setup and highlights that C9 engages a complementary but non-identical microenvironment compared with the reference ligand.

**TABLE 10:** Details of chemical interaction of co-crystalized compound with KRAS G12 binding pocket.

Name	XYZ:X	XYZ:Y	XYZ:Z	Distance	Category	From	From chemistry	To	To chemistry
A:ARG161:NH1 - N:UNK1:O	−2.3605	32.5235	35.009	5.55825	Electrostatic	A:ARG161:NH1	Positive	N:UNK1:O	Negative
A:ARG161:NH1 - N:UNK1:O	−2.1455	34.5075	36.2335	3.11101	Electrostatic	A:ARG161:NH1	Positive	N:UNK1:O	Negative
A:ARG161:NH1 - N:UNK1:O	−3.747	34.559	34.565	4.93326	Electrostatic	A:ARG161:NH1	Positive	N:UNK1:O	Negative
A:THR158:HG1 - N:UNK1:O	−2.6095	30.1995	34.149	2.29217	Hydrogen bond	A:THR158:HG1	H-donor	N:UNK1:O	H-acceptor
A:ARG161:HH11 - N:UNK1:O	−3.2095	35.3805	34.6405	2.50646	Hydrogen bond	A:ARG161:HH11	H-donor	N:UNK1:O	H-acceptor
A:ARG161:HH12 - N:UNK1:O	−1.185	33.2155	35.7535	2.47248	Hydrogen bond	A:ARG161:HH12	H-donor	N:UNK1:O	H-acceptor
A:ARG161:HH21 - N:UNK1:O	−3.447	36.4505	34.989	2.65184	Hydrogen bond	A:ARG161:HH21	H-donor	N:UNK1:O	H-acceptor
N:UNK1:H - A:ASP154:OD1	−3.5245	32.516	33.337	2.46273	Hydrogen bond	N:UNK1:H	H-donor	A:ASP154:OD1	H-acceptor
N:UNK1:H - A:THR158:OG1	−2.22	32.1705	33.8875	2.7364	Hydrogen bond	N:UNK1:H	H-donor	A:THR158:OG1	H-acceptor
N:UNK1:H - N:UNK1:O	−5.987	31.8555	36.4525	2.8911	Hydrogen bond	N:UNK1:H	H-donor	N:UNK1:O	H-acceptor
A:ALA134:C - N:UNK1:O	−0.1825	19.1655	37.06	3.35859	Hydrogen bond	A:ALA134:C	H-donor	N:UNK1:O	H-acceptor
A:ARG161:CD - N:UNK1:O	−0.5445	33.4775	36.7565	3.30101	Hydrogen bond	A:ARG161:CD	H-donor	N:UNK1:O	H-acceptor
N:UNK1 - A:ALA134	−1.68033	20.2327	35.3445	4.16894	Hydrophobic	N:UNK1	Pi-orbitals	A:ALA134	Alkyl

Our integrated workflow—pairing an interpretable GA-MLR with robust validation and orthogonal docking—argues that C9 is a credible KRAS^G12C Switch-II pocket (SII-P) binder while clarifying where the model’s predictions are most reliable. We deliberately prioritize explainability and calibrated uncertainty over marginal gains in point accuracy, because mechanistic hypotheses and decision-grade error bars are the levers that most improve prospective medicinal chemistry on small, congeneric datasets.

In the fitted GA-MLR, the dominant descriptor families point to two complementary physical themes with direct design implications for Switch-II pocket (SII-P) engagement. First, 3D distribution descriptors (RDF/MoRSE, with different atomic weightings) capture how mass, volume, and electronegativity are arranged at specific distance shells; the positive coefficients at selected shells imply that compact hydrophobic density and appropriately placed polarity at those radii favor activity—consistent with burying non-polar surface under the SII-P “lid” while projecting donors/acceptors toward the H95–Y96–Q99 cryptic subpocket exploited by KRAS^G12C covalent chemotypes (Lanman et al., 2019; Ostrem et al., 2013; Canon et al., 2019; Fell et al., 2020; Schuur et al., 1996; Hemmer et al., 1999; Gramatica, 2020). Second, surface-partition terms (e.g., fractional positive polar surface area, FPSA) weight where chargeable/heteroatom surface is exposed; their positive sign supports moderated, localized polarity rather than wholesale charge—compatible with known permeability/recognition trade-offs and with the hydrogen-bond topology reported for SII-P binders (Lanman et al., 2019; Ostrem et al., 2013; Canon et al., 2019; Fell et al., 2020; Ertl et al., 2000). By contrast, global exposure metrics (e.g., TASA) trending negative argue against over-extended solvent-facing area, reinforcing a design bias toward tighter shape complementarity and fewer, better polar contacts. Practically, these signals recommend (i) increasing lipophilic bulk along vectors that deepen contact in the Y96/Q99 wall, (ii) retaining a focused donor/acceptor pattern aligned to the observed pose, and (iii) avoiding gratuitous polar surface that would inflate TASA without productive pocket interactions—together offering a descriptor-anchored blueprint for the next round of analogs (Sung et al., 2021; Pylayeva-Gupta et al., 2011; Westover et al., 2016; Cox et al., 2014; Tropsha, 2010; Cherkasov et al., 2014; Todeschini and Consonni, 2009; Lanman et al., 2019; Cao et al., 2013; Sulaiman et al., 2021; C et al., 2002; Ghani and Ahmad, 2010; Molnar et al., 2018; Roy et al., 2015; López-López et al., 2019; Ostrem et al., 2013; Canon et al., 2019; Fell et al., 2020; Schuur et al., 1996; Hemmer et al., 1999; Gramatica, 2020).

Although higher-capacity baselines (PLS, RF) can outperform linear models on a given split, GA-MLR remains the most useful engine for hypothesis generation: its coefficients map cleanly onto physically interpretable descriptor families (vdW/shape-weighted RDF/MoRSE; balanced positive polar surface area), yielding actionable guidance for substituent placement and polarity tuning within SII-P. The dispersion observed in repeated cross-validation is a faithful readout of the small-N regime rather than a model defect, and Y-randomization effectively rules out chance correlations. Together with split-conformal prediction intervals, which provide calibrated coverage, the framework supplies both rank ordering and uncertainty—information that is more actionable than point estimates alone when advancing compounds into synthesis and testing.

Generalizability is constrained, by design, to a single congeneric covalent series. Applicability-domain (AD) analysis indicates that most training analogs lie within the modeled subspace, whereas a subset of test compounds fall outside; we therefore treat inside-AD predictions as decision-preferred and outside-AD as hypothesis-generating. Leave-cluster-out validation, which withholds whole descriptor neighborhoods, further stresses the model and exposes performance heterogeneity typical of narrow chemical neighborhoods. In this context, reporting both AD stratification and conformal intervals makes explicit the reliability envelope for any given prediction.

Docking provides orthogonal structural support. The C9 pose occupies the canonical SII-P cavity and projects toward the H95–Y96–Q99 cryptic subpocket that underpinned the transformation of KRAS^G12C from “undruggable” concept to a clinically validated target, beginning with allosteric covalent inhibitors that lock the GDP state and culminating in sotorasib (AMG-510) and adagrasib (MRTX849) (Lanman et al., 2019; Ostrem et al., 2013; Canon et al., 2019; Fell et al., 2020). This atomistic picture coheres with the GA-MLR descriptor signals—hydrophobic packing and moderated polarity at specific 3D radii—reinforcing that the linear model is capturing pocket physics rather than spurious correlations. We interpret the −9.6 kcal/mol docking score qualitatively, consistent with community benchmarks that docking is most reliable for pose generation and less so for precise affinity ranking (Warren et al., 2006; Huang and Zou, 2010). Moreover, noncovalent docking does not explicitly model the G12C reaction coordinate, and SII-P conformational plasticity (cryptic breathing, water networks) can modulate recognition; covalent docking and short explicit-water molecular dynamics are therefore logical next steps to stress-test pose stability and reactive geometry in a pocket known to be dynamic (Huang and Zou, 2010; Mou et al., 2025).

Finally, the evidence positions C9 as a mechanistically rational KRAS^G12C candidate: an SII-P-consistent pose aligned with descriptor-level SAR, decision-grade uncertainty quantification, and clear AD guidance for triaging experiments. Practically, the data motivate a staged plan: biochemical engagement and nucleotide-exchange assays for KRAS^G12C, orthogonal biophysics (e.g., intact-protein MS for covalent adducts; DSF/CETSA), and comparative docking/short MD (including covalent protocols) against sotorasib/adagrasib-like matter to refine vectors that deepen H95/Y96/Q99 engagement. Positive outcomes would justify cellular studies in KRAS^G12C-mutant models and inform subsequent lead-optimization cycles. Within the realistic constraints of a small, single-series dataset, this balance—interpretability, calibration, and structural plausibility—maximizes the likelihood of successful translation from *in silico* predictions to *in vitro* validation.

Therefore, this study presents a novel pipeline that synergizes interpretable machine learning with evolution-based molecular generation, yielding compound C9 as a promising KRAS inhibitor with predictive potency, safety, and structural validity. The ability to pinpoint such candidates within an interpretable and chemically meaningful framework holds substantial promise for guiding experimental validation and future lead optimization efforts against KRAS-driven malignancies.

## Conclusion

This study demonstrates the successful integration of QSAR modeling and *de novo* design to identify novel KRAS inhibitors with strong predicted potency and favorable drug-like properties. Among the models developed, PLS and Random Forest offered high predictive accuracy, while the GA-MLR model provided a mechanistically interpretable equation based on eight key molecular descriptors. Virtual screening of 58 *de novo* compounds identified compound C9 as a potent, non-toxic candidate within the model’s applicability domain, highlighting its potential as a lead structure for further development in lung cancer therapy. These findings offer a promising computational pipeline for accelerating structure-based drug discovery against challenging oncogenic targets like KRAS.

## Data Availability

The original contributions presented in the study are included in the article/Supplementary Material, further inquiries can be directed to the corresponding author.

## Abbreviations

NSCLC, Non-small cell lung cancer; KRAS, Kirsten rat sarcoma viral oncogene homolog; QSAR, Quantitative structure–activity relationship; MLR, Multiple linear regression; GA, Genetic Algorithm; GA-MLR, Genetic Algorithm-optimized Multiple Linear Regression; AIC, Akaike Information Criterion; PLS, Partial Least Squares; RF, Random Forest; MSE, Mean squared error; XGBoost, Extreme Gradient Boosting; RMSE, Root mean square error; MAE, Mean absolute error; Grav, gravitational index; TASA, Total solvent-accessible surface area.
