# Supplementary figures and images for: QSAR-guided discovery of novel KRAS inhibitors for lung cancer therapy
Source: Front Bioinform. 2025 Nov 17;5:1663846. doi: 10.3389/fbinf.2025.1663846 (PMC12665777; doi:10.3389/fbinf.2025.1663846)

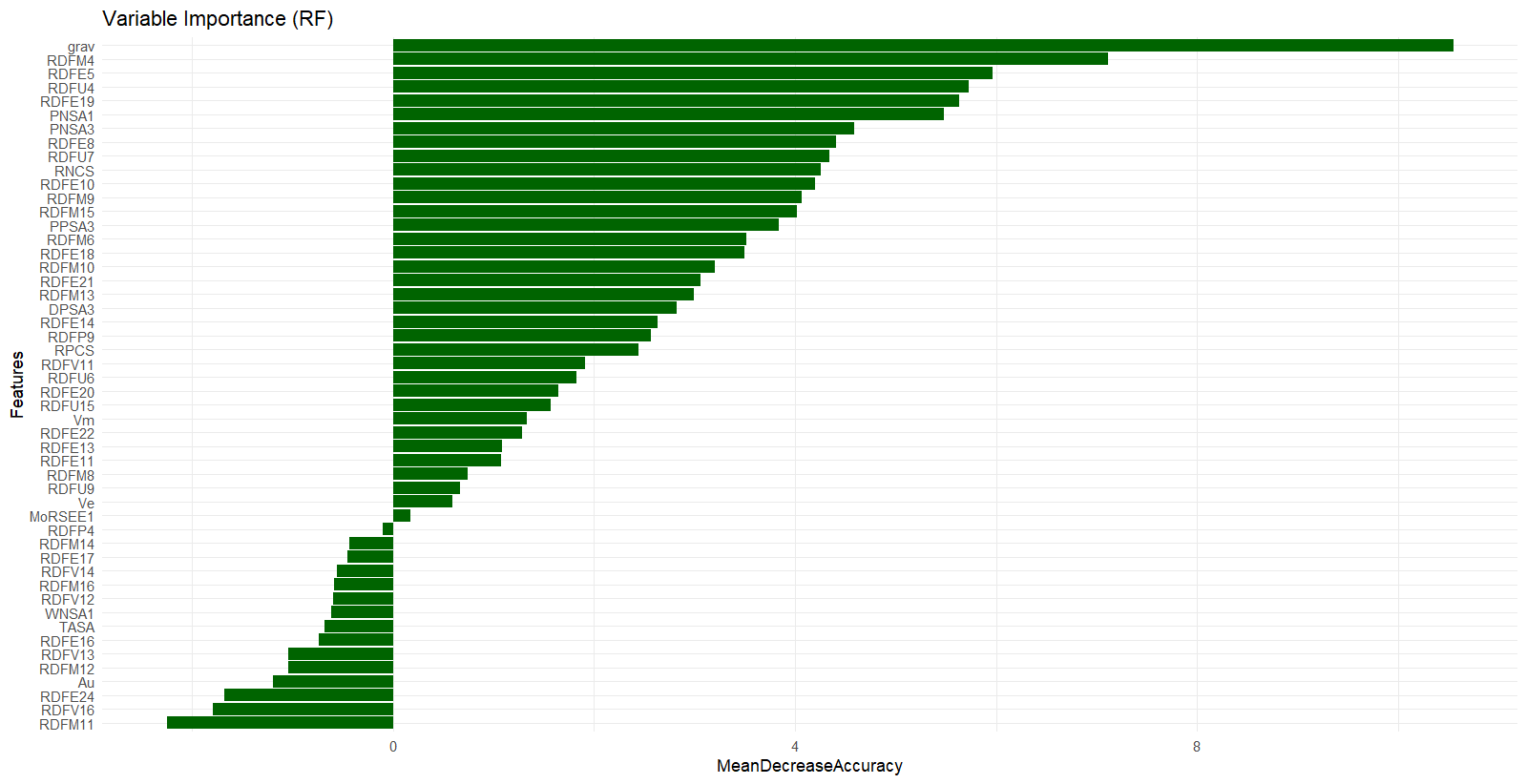


Figure S1

Supplement: Supplementary file 3 [file Supplementaryfile1.docx]
