# Supplementary material for: QSAR-guided discovery of novel KRAS inhibitors for lung cancer therapy
Source: Front Bioinform. 2025 Nov 17;5:1663846. doi: 10.3389/fbinf.2025.1663846 (PMC12665777; doi:10.3389/fbinf.2025.1663846)
Supplement: Supplementary file 6 [file Supplementaryfile2.docx]

# ====================== 1. Load Required Libraries ======================

libs <- c("caret", "randomForest", "xgboost", "Metrics", "ggplot2", "corrplot", "pls",

"GA", "iml", "partykit", "kableExtra", "dplyr", "tidyr", "tibble")

invisible(lapply(libs, library, character.only = TRUE))

set.seed(123)

# ====================== 2. Simulate Dataset ======================

n <- 100

sim_data <- data.frame(

Compound_ID = paste0("Cmpd_", 1:n),

MW = rnorm(n, 450, 50),

logP = rnorm(n, 3.5, 1.0),

HBA = rpois(n, 6),

HBD = rpois(n, 2),

TPSA = rnorm(n, 90, 25),

RB = rpois(n, 8),

AromaticRings = rpois(n, 3),

AliphaticRings = rpois(n, 2),

HeavyAtoms = rnorm(n, 30, 3),

Complexity = rnorm(n, 550, 100),

IC50 = 10^runif(n, -1, 3),

Ki = 10^runif(n, -1, 3)

)

sim_data$pIC50 <- -log10(sim_data$IC50 * 1e-9)

sim_data$pKi <- -log10(sim_data$Ki * 1e-9)

write.csv(sim_data, "simulated_kras_dataset.csv", row.names = FALSE)

# ====== Load and Preprocess Data ======

library(caret)

library(GA)

df <- read.csv("Chemopy result.csv")

df <- df[, sapply(df, is.numeric)] # keep only numeric columns

df <- df[complete.cases(df), ] # remove rows with NA

target <- "pIC50"

features <- setdiff(names(df), c("IC50", "Ki", "pKi", target))

df_model <- df[, c(features, target)]

# Train-test split

set.seed(123)

split <- createDataPartition(df_model[[target]], p = 0.7, list = FALSE)

train_data <- df_model[split, ]

test_data <- df_model[-split, ]

X_train <- train_data[, features]

X_test <- test_data[, features]

# ====== Dimensionality Reduction ======

# Remove highly correlated features

cor_matrix <- cor(X_train)

high_corr <- findCorrelation(cor_matrix, cutoff = 0.95)

X_train <- X_train[, -high_corr]

X_test <- X_test[, -high_corr]

# Select top 50 most variable features

top_var_idx <- order(apply(X_train, 2, var), decreasing = TRUE)[1:50]

X_train <- X_train[, top_var_idx]

X_test <- X_test[, top_var_idx]

# Normalize

preproc <- preProcess(X_train, method = c("center", "scale"))

X_train <- predict(preproc, X_train)

X_test <- predict(preproc, X_test)

# Update training data

train_data <- cbind(X_train, pIC50 = train_data[[target]])

test_data <- cbind(X_test, pIC50 = test_data[[target]])

y_test <- test_data[[target]] # define y_test

# ====== GA Feature Selection Function ======

fitness_func <- function(vars) {

selected <- which(vars == 1)

if (length(selected) < 2) return(0)

selected_features <- colnames(X_train)[selected]

formula <- as.formula(paste(target, "~", paste(selected_features, collapse = "+")))

result <- tryCatch({

model <- lm(formula, data = train_data)

adj_r2 <- summary(model)$adj.r.squared

penalty <- length(selected) / nrow(train_data)

score <- adj_r2 - penalty

if (is.na(score) || is.nan(score)) return(0)

return(score)

}, error = function(e) return(0))

return(result)

}

# ====== Run Genetic Algorithm ======

set.seed(123)

ga_result <- ga(

type = "binary",

fitness = fitness_func,

nBits = ncol(X_train),

maxiter = 50,

run = 10

)

ga_features <- colnames(X_train)[which(ga_result@solution[1,] == 1)]

train_ga <- train_data[, c(ga_features, target)]

test_ga <- test_data[, c(ga_features, target)]

# ====== GA-MLR Model ======

mlr_model <- lm(as.formula(paste(target, "~", paste(ga_features, collapse = "+"))), data = train_ga)

mlr_pred <- predict(mlr_model, newdata = test_ga)

mlr_perf <- postResample(mlr_pred, test_ga[[target]])

# ====== Stepwise MLR ======

full_model <- lm(pIC50 ~ ., data = train_data)

null_model <- lm(pIC50 ~ 1, data = train_data)

stepwise_model <- stats::step(null_model, scope = list(lower = null_model, upper = full_model),

direction = "both", trace = FALSE)

mlr_pred_stepwise <- predict(stepwise_model, newdata = test_data)

mlr_perf_stepwise <- postResample(mlr_pred_stepwise, y_test)

# ====== Output Performance ======

print("GA-MLR Performance:")

print(mlr_perf)

print("Stepwise MLR Performance:")

print(mlr_perf_stepwise)

# ====================== 6. Other Models ======================

# --- PLS ---

pls_model <- plsr(pIC50 ~ ., data = train_data, validation = "CV")

pls_pred <- predict(pls_model, newdata = test_data, ncomp = 2)[,,1]

pls_perf <- postResample(pls_pred, y_test)

# --- Random Forest ---

rf_model <- randomForest(pIC50 ~ ., data = train_data, ntree = 500, importance = TRUE)

rf_pred <- predict(rf_model, test_data)

rf_perf <- postResample(rf_pred, y_test)

# ==== XGBoost Model ====

library(xgboost)

# Use only GA-selected features (same as used in mlr_model)

xgb_train <- xgb.DMatrix(data = as.matrix(train_ga[, ga_features]), label = train_ga[[target]])

xgb_test <- xgb.DMatrix(data = as.matrix(test_ga[, ga_features]), label = test_ga[[target]])

# Train XGBoost model

xgb_model <- xgboost(data = xgb_train,

objective = "reg:squarederror",

nrounds = 200,

max.depth = 6,

eta = 0.1,

verbose = 0)

# Predict

xgb_pred <- predict(xgb_model, xgb_test)

# Evaluate

xgb_perf <- postResample(xgb_pred, test_ga[[target]])

print(xgb_perf)

# ====================== 7. Model Comparison Table ======================

model_summary <- tibble(

Model = c("GA-MLR", "Stepwise MLR", "PLS", "Random Forest", "XGBoost"),

R_squared = c(mlr_perf["Rsquared"], mlr_perf_stepwise["Rsquared"],

pls_perf["Rsquared"], rf_perf["Rsquared"], xgb_perf["Rsquared"]),

RMSE = c(mlr_perf["RMSE"], mlr_perf_stepwise["RMSE"],

pls_perf["RMSE"], rf_perf["RMSE"], xgb_perf["RMSE"])

)

kable(model_summary, caption = "Model Validation Metrics", digits = 3) %>%

kable_styling(bootstrap_options = c("striped", "hover", "condensed"))

write.csv(model_summary, "KRAS_Model_Comparison.csv", row.names = FALSE)

# ====================== 8. SHAP-like Interpretation ======================

# Refit RF model using current valid GA features

rf_model_ga <- randomForest(

formula = as.formula(paste(target, "~", paste(ga_features, collapse = "+"))),

data = train_ga,

ntree = 500

)

# Now build Predictor with the refitted model

predictor_rf <- iml::Predictor$new(

model = rf_model_ga,

data = train_ga[, ga_features],

y = train_ga[[target]]

)

# Compute SHAP values for the first observation

shap_rf <- Shapley$new(predictor_rf, x.interest = train_ga[1, ga_features])

library(ggplot2)

# Extract Shapley result data

shap_df <- shap_rf$results

# Optional: keep top contributing features

top_shap <- shap_df[order(abs(shap_df$phi), decreasing = TRUE), ][1:15, ]

# Plot

ggplot(top_shap, aes(x = reorder(feature, phi), y = phi, fill = phi > 0)) +

geom_bar(stat = "identity") +

coord_flip() +

theme_minimal(base_size = 14) +

labs(

title = "Top 15 SHAP Feature Contributions (Random Forest)",

x = "Feature",

y = "SHAP Value (phi)"

) +

scale_fill_manual(values = c("TRUE" = "steelblue", "FALSE" = "firebrick")) +

theme(legend.position = "none")

write.csv(shap_rf$results, "shap_rf_contributions.csv", row.names = FALSE)

feat_imp_rf <- FeatureImp$new(predictor_rf, loss = "mse")

library(ggplot2)

# Extract and sort top 15 features by importance

imp_df <- feat_imp_rf$results

top_imp <- imp_df[order(imp_df$importance, decreasing = TRUE), ][1:15, ]

# Plot

ggplot(top_imp, aes(x = reorder(feature, importance), y = importance)) +

geom_bar(stat = "identity", fill = "darkgreen") +

geom_errorbar(aes(ymin = importance.05, ymax = importance.95), width = 0.3) +

coord_flip() +

theme_minimal(base_size = 14) +

labs(

title = "Top 15 Feature Importances (Permutation-Based, RF Model)",

x = "Feature",

y = "Importance (Increase in MSE)"

)

write.csv(feat_imp_rf$results, "rf_permutation_importance.csv", row.names = FALSE)

# ====================== 9. Visualizations ======================

# Residual Plot

ggplot(data.frame(True = test_data[[target]], Pred = rf_pred), aes(x = True, y = Pred)) +

geom_point(color = "blue") +

geom_abline(slope = 1, intercept = 0, linetype = "dashed", color = "red") +

theme_minimal() +

labs(title = "Random Forest: Observed vs Predicted", x = "True pIC50", y = "Predicted pIC50")

# Variable Importance (RF)

imp_df <- data.frame(Feature = rownames(importance(rf_model)),

Importance = importance(rf_model)[,1])

ggplot(imp_df, aes(x = reorder(Feature, Importance), y = Importance)) +

geom_bar(stat = "identity", fill = "darkgreen") +

coord_flip() +

theme_minimal() +

labs(title = "Variable Importance (RF)", x = "Features", y = "Mean Decrease Accuracy")

write.csv(imp_df, "KRAS_RF_Importance.csv", row.names = FALSE)

# ====================== 10. Compare GA vs Stepwise Coefficients ======================

coef_ga <- coef(summary(mlr_model)) %>%

as.data.frame() %>%

tibble::rownames_to_column("Variable") %>%

dplyr::rename(Estimate_GA = Estimate, p_GA = `Pr(>|t|)`)

coef_stepwise <- coef(summary(stepwise_model)) %>%

as.data.frame() %>%

tibble::rownames_to_column("Variable") %>%

dplyr::rename(Estimate_Stepwise = Estimate, p_Stepwise = `Pr(>|t|)`)

merged_coef <- full_join(

coef_ga[, c("Variable", "Estimate_GA", "p_GA")],

coef_stepwise[, c("Variable", "Estimate_Stepwise", "p_Stepwise")],

by = "Variable"

)

kable(merged_coef, digits = 4, caption = "Comparison of GA vs Stepwise MLR Coefficients") %>%

kable_styling(bootstrap_options = c("striped", "hover", "condensed"))

write.csv(merged_coef, "GA_vs_Stepwise_MLR_Coefficients.csv", row.names = FALSE)

# ==== 1. GA-Selected MLR Equation ====

mlr_model <- lm(as.formula(paste(target, "~", paste(ga_features, collapse = "+"))), data = train_ga)

library(stargazer)

# Show equation

summary(mlr_model) # Publication-ready table

stargazer::stargazer(mlr_model, type = "text") # Optional clean table

# ==== 2. Predictions ====

mlr_pred_train <- predict(mlr_model, newdata = train_ga)

mlr_pred_test <- predict(mlr_model, newdata = test_ga)

# ==== 3. Validation Metrics ====

mlr_perf_train <- postResample(mlr_pred_train, train_ga[[target]])

mlr_perf_test <- postResample(mlr_pred_test, test_ga[[target]])

mlr_metrics <- tibble(

Dataset = c("Train", "Test"),

R_squared = c(mlr_perf_train["Rsquared"], mlr_perf_test["Rsquared"]),

RMSE = c(mlr_perf_train["RMSE"], mlr_perf_test["RMSE"]),

MAE = c(mae(train_ga[[target]], mlr_pred_train), mae(test_ga[[target]], mlr_pred_test))

)

kable(mlr_metrics, caption = "GA-MLR Performance Metrics") %>%

kable_styling(bootstrap_options = c("striped", "hover"))

# ==== 4. Observed vs Predicted Plot ====

obs_pred_plot <- function(obs, pred, title) {

ggplot(data.frame(Observed = obs, Predicted = pred), aes(x = Observed, y = Predicted)) +

geom_point(color = "blue", alpha = 0.7) +

geom_abline(slope = 1, intercept = 0, linetype = "dashed", color = "red") +

theme_minimal() +

labs(title = title, x = "Observed pIC50", y = "Predicted pIC50")

}

# Combine train and test predictions

df_obs_pred <- bind_rows(

data.frame(Set = "Train", Observed = train_ga[[target]], Predicted = mlr_pred_train),

data.frame(Set = "Test", Observed = test_ga[[target]], Predicted = mlr_pred_test)

)

# Plot with legend

ggplot(df_obs_pred, aes(x = Observed, y = Predicted, color = Set, shape = Set)) +

geom_point(size = 3, alpha = 0.7) +

geom_abline(slope = 1, intercept = 0, linetype = "dashed", color = "black") +

theme_minimal() +

labs(

title = "GA-MLR: Observed vs Predicted pIC50",

x = "Observed pIC50",

y = "Predicted pIC50"

) +

theme(legend.title = element_blank())

# ==== 5. Coefficient Plot ====

mlr_coef <- coef(summary(mlr_model)) %>%

as.data.frame() %>%

tibble::rownames_to_column("Variable")

ggplot(mlr_coef, aes(x = reorder(Variable, Estimate), y = Estimate)) +

geom_bar(stat = "identity", fill = "steelblue") +

coord_flip() + theme_minimal() +

labs(title = "GA-MLR Coefficients", x = "Variable", y = "Estimate")

# ====== Load and Preprocess Data ======

library(caret)

library(GA)

df <- read.csv("Chemopy result.csv")

df <- df[, sapply(df, is.numeric)] # keep only numeric columns

df <- df[complete.cases(df), ] # remove rows with NA

target <- "pIC50"

features <- setdiff(names(df), c("IC50", "Ki", "pKi", target))

df_model <- df[, c(features, target)]

# Train-test split

set.seed(123)

split <- createDataPartition(df_model[[target]], p = 0.7, list = FALSE)

train_data <- df_model[split, ]

test_data <- df_model[-split, ]

X_train <- train_data[, features]

X_test <- test_data[, features]

# === Use pre-normalized X_train before applying preProcess() ===

X_train_raw <- train_data[, ga_features] # This is before centering/scaling

# Get raw means and SDs for scaling new data

ga_means_raw <- sapply(X_train_raw, mean)

ga_sds_raw <- sapply(X_train_raw, sd)

# Combine and export

norm_stats_raw <- data.frame(

Feature = names(ga_means_raw),

Mean = round(ga_means_raw, 5),

SD = round(ga_sds_raw, 5)

)

# Save for use in screening

write.csv(norm_stats_raw, "GA_Feature_Normalization_Stats_RAW.csv", row.names = FALSE)

# Named vectors for scaling new data

mean_vector <- setNames(norm_stats_raw$Mean, norm_stats_raw$Feature)

sd_vector <- setNames(norm_stats_raw$SD, norm_stats_raw$Feature)
